# Neurological, Psychiatric, and Psychological Implications of the COVID-19 Pandemic: Protocol for a Large-Scale Umbrella Review of Observational Studies

**DOI:** 10.3390/ijerph19031681

**Published:** 2022-02-01

**Authors:** Ta-Chuan Yeh, Chih-Sung Liang, Chia-Kuang Tsai, Marco Solmi, Beny Lafer, Ping-Tao Tseng, Chih-Wei Hsu, Pao-Yen Lin, Joseph Firth, Brendon Stubbs, Lamiece Hassan, Michele Fornaro, Eduard Vieta, Trevor Thompson, Jaeil Shin, Andre F. Carvalho

**Affiliations:** 1 National Defense Medical Center, Department of Psychiatry, Tri-Service General Hospital, Taipei 114, Taiwan; fantine7520@ndmctsgh.edu.tw; 2 Department of Psychiatry, Penghu Branch, Tri-Service General Hospital, Penghu 880, Taiwan; 3 Institute of Brain Science, National Yang Ming Chiao Tung University, Taipei 112, Taiwan; 4 Department of Psychiatry, Beitou Branch, Tri-Service General Hospital, National Defense Medical Center, Taipei 112, Taiwan; 5 Department of Neurology, Tri-Service General Hospital, National Defense Medical Center, Taipei 114, Taiwan; 6 Department of Psychiatry, University of Ottawa, Ottawa, ON K1Z 7K4, Canada; marco.solmi83@gmail.com; 7 Department of Mental Health, The Ottawa Hospital, Ottawa, ON K1Z 7K4, Canada; 8 Ottawa Hospital Research Institute (OHRI), Clinical Epidemiology Program, University of Ottawa, Ottawa, ON K1Z 7K4, Canada; 9 Bipolar Research Program, Department and Institute of Psychiatry, University of São Paulo Medical School, São Paulo 05403-903, Brazil; benylafer@gmail.com; 10 Prospect Clinic for Otorhinolaryngology & Neurology, Kaohsiung 811, Taiwan; ducktseng@gmail.com; 11 Institute of Biomedical Sciences, National Sun Yat-sen University, Kaohsiung 804, Taiwan; 12 Department of Psychology, College of Medical and Health Science, Asia University, Taichung 413, Taiwan; 13 Department of Psychiatry, Kaohsiung Chang Gung Memorial Hospital, Chang Gung University College of Medicine, Kaohsiung 833, Taiwan; harwicacademia@gmail.com (C.-W.H.); paoyenlin@gmail.com (P.-Y.L.); 14 Department of Computer Science and Information Engineering, National Cheng Kung University, Tainan 701, Taiwan; 15 Institute for Translational Research in Biomedical Sciences, Kaohsiung Chang Gung Memorial Hospital, Kaohsiung 833, Taiwan; 16 Division of Psychology and Mental Health, Manchester Academic Health Science Centre, The University of Manchester, Manchester M13 9PL, UK; joseph.firth@manchester.ac.uk (J.F.); lamiece.hassan@manchester.ac.uk (L.H.); 17 Physiotherapy Department, South London and Maudsley NHS Foundation Trust, London SE13 6QJ, UK; brendon.stubbs@kcl.ac.uk; 18 Department of Psychological Medicine, Institute of Psychiatry, Psychology and Neuroscience (IoPPN), King’s College London, London SE5 8AF, UK; 19 Section of Psychiatry, Department of Neuroscience, Reproductive Science, and Dentistry, Federico II University of Naples, 80138 Naples, Italy; dott.fornaro@gmail.com; 20 Bipolar and Depressive Disorders Unit, Institute of Neuroscience, Hospital Clinic, University of Barcelona, 08035 Barcelona, Spain; evieta@clinic.cat; 21 Centre for Chronic Illness and Ageing, University of Greenwich, London SE10 9LS, UK; t.thompson@greenwich.ac.uk; 22 Department of Pediatrics, Yonsei University College of Medicine, Seoul 03722, Korea; shinji@yuhs.ac; 23 Innovation in Mental and Physical Health and Clinical Treatment Strategic Research Centre, School of Medicine, Barwon Health, Deakin University, Geelong, VIC 3220, Australia; andrefc7@hotmail.com

**Keywords:** COVID-19, SARS-CoV-2, pandemic, mental health, nervous system diseases, psychiatric diseases

## Abstract

The severe acute respiratory syndrome coronavirus 2 disease (SARS-CoV-2) is the most severe manifestation of the coronavirus disease 2019 (COVID-19) pandemic. Accruing evidence indicates that the COVID-19 pandemic may have profound deleterious neurological, psychiatric, and psychological outcomes. The number of systematic reviews (SRs) and meta-analyses (MAs) on this topic has grown exponentially. This protocol aims to synthesize all evidence from SRs and MAs on the associations between the COVID-19 pandemic and neuropsychiatric outcomes. The following electronic databases will be systematically searched from inception up to 15 January 2022: PubMed, Embase, APA PsycINFO, and Cochrane Reviews. An umbrella review (UR) of SRs and MAs of observational studies will be conducted. SRs and/or MAs of observational studies examining any direct or indirect association of COVID-19 with the neuropsychiatric outcomes will be deemed eligible for potential inclusion in this UR. The direct associations include the impact on the (1) prognosis of COVID-19 and (2) neuropsychiatric sequelae after COVID-19 infection. The indirect associations include the influence of the COVID-19 pandemic on the (1) treatments and (2) outcomes of neurological and psychiatric conditions associated with the COVID-19 pandemic.

## 1. Introduction

In December 2019, the world witnessed the emergence of the severe acute respiratory syndrome coronavirus 2 (SARS-CoV-2) infection, which posed an extraordinary threat to global public health and human safety [1]. SARS-CoV-2, a highly contagious and pathogenic virus, rapidly disseminated across the world, causing a pandemic of coronavirus disease 2019 (COVID-19). In addition to pulmonary pathology, COVID-19 is now recognized as a systemic disease associated with a broad spectrum of manifestations (e.g., hematological, cardiovascular, renal, and neuropsychiatric) [2,3,4]. The mechanisms driving multi-organ damage may involve direct viral infection and toxicity, endothelial cell damage, dysregulated immune response, cytokine storm, and maladaptive functions of the renin-angiotensin-aldosterone system [3,4]. Moreover, accumulating evidence suggests persistent and prolonged effects on multiple organs and the brain after the acute COVID-19 subsides [2,5,6,7,8].

The COVID-19 pandemic presents unprecedented challenges to the lives of people around the world. It has caused devastating impacts on livelihood, society, health care systems, and economic activity [9,10]. Major stressors may include social restrictions, lockdowns, boredom, loneliness, infection fears, conflicting messages from authorities, inadequate supplies, financial loss, stigma, and shifting priorities of governments in their attempt to control COVID-19 outbreaks [9,10,11,12]. The utilization of healthcare services may also be affected because of measures such as stay-at-home orders and lockdowns [9,11,12]. As a consequence of the devastating impact of the COVID-19 pandemic at all these levels, evidence has indicated a meaningful and deleterious mental health impact on populations worldwide [9,11,12]. For example, a global study reported an increase of 27.6% and 25.6%, in the prevalence of major depressive disorder and anxiety disorders, respectively, since before the pandemic [13].

Though the clinical presentation of the SARS-CoV-2 infection is mainly associated with cardiac or pulmonary complications, it has been reported that coronaviruses may also invade the central and peripheral nervous systems, inducing neurological and psychiatric symptoms. According to a previous comprehensive review, COVID-19 infected patients were more likely to demonstrate a variety of neurological pathologies, such as microgliosis (52.5%), astrogliosis (45.6%), inflammatory infiltrates (44.0%), hypoxic-ischemic lesions (40.8%), edema (25.3%), and hemorrhagic lesions (20.5%) [14]. Importantly, several meta-analyses have indicated that health care workers (HCWs) working in pandemics are at increased risk of a range psychiatric symptoms. Among HCWs, the overall prevalence of generalized anxiety disorder was 30.5% [15], prevalence of acute ost-traumatic stress disorder ranged from 25.1% to 71.5% [16], and prevalence of depression was about 20.2% [17].

The literature has also witnessed an increase in the number of published systematic reviews (SRs) and meta-analyses (MAs) related to possible neurological, psychiatric, and psychological implications of the COVID-19 pandemic [18,19]; however, concerns have been raised regarding the methodological quality of published SRs and MAs related to the COVID-19 pandemic [20]. An evidence mapping included 243 systematic reviews about COVID-19 infection and reported that 25.9% were of low quality and 61.7% were of critically low quality [20]. An umbrella review is a knowledge synthesis approach that enables a quality-based assessment of the available evidence derived from SRs and MAs on a given topic of interest [21]. Therefore, we propose to undertake an umbrella review to summarize and synthesize evidence from observational studies reporting on bidirectional associations of the COVID-19 infection with neurological, psychiatric, and psychological outcomes in a direct or indirect manner.

## 2. Materials and Methods

**Figure 1:**
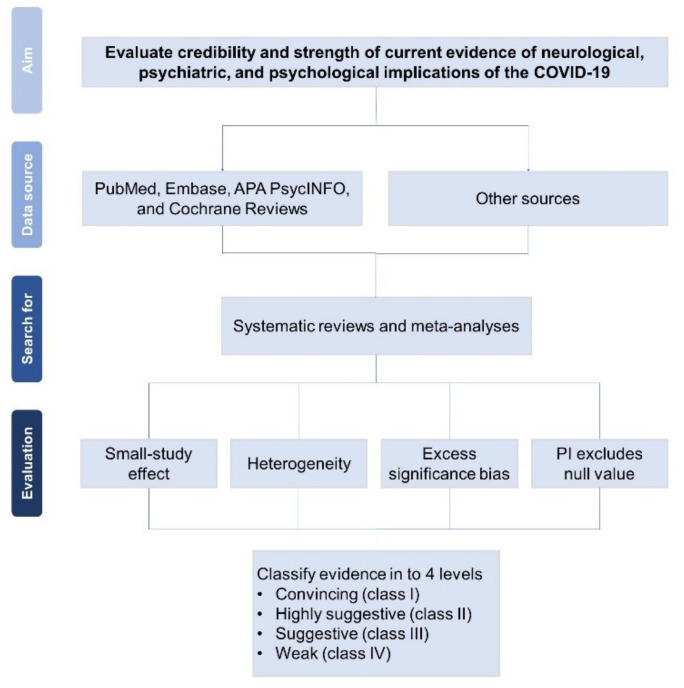
Flowchart of study process. PI = prediction interval.

This umbrella review follows methods that were previously used in another similar knowledge synthesis [22]. The PubMed, Embase, APA PsycINFO, and Cochrane Reviews computerized databases will be systematically searched from inception to 15 January 2022, with no date or language restrictions, to identify SRs and MAs on the associations between COVID-19 and neurological, psychiatric, and psychological outcomes. The protocol of umbrella review follows the of Preferred Reporting Items for Systematic Review and Meta-analysis Protocols (PRISMA-P) [23]. The final results of this study will be reported according to PRISMA [24]. Figure 1 shows the study process.

### 2.1. Search Strategy

A search strategy was built by a professional librarian (Risa Shorr; The Ottawa Hospital, Ottawa, Ontario, Canada) and is available as a supplementary online material. According to the indices of various databases, the following keywords will be used with Boolean operators: “COVID-19”, “Coronavirus Disease 2019”, “new coronavirus”, “2019-nCoV”, “novel coronavirus”, “SARS-CoV-2”, “pandemic” and “mental disorders”, “psychiatric disorders”, “neurological disorders”, “nervous system diseases”, “brain disorders”, “psychological phenomena”, “systematic review” or “meta-analysis” on PubMed, EMBASE, APA PsycINFO, and the Cochrane Reviews. Two investigators with considerable clinical research experience and expertise in the field of the umbrella review independently screen the titles and abstracts of retrieved references for eligibility. Duplicated articles will be removed. The full text of the remaining articles will be read and reference lists of these papers will be searched for additional relevant articles. These are reviewed for inclusion using the same method as articles found through database searches. Disagreements are resolved by consensus or via discussion with a third investigator.

### 2.2. Inclusion Criteria

We include SRs and MAs focusing on the direct or indirect associations of COVID-19 with neurological, psychological, and psychiatric outcomes. The direct associations may include (1) neurological, psychiatric, and psychological sequelae due to COVID-19 infection (e.g., olfactory dysfunction, delirium, anxiety, depression) and (2) the impact of neurological and psychiatric disorders as well as psychological factors on the prognosis of COVID-19 (e.g., mortality). The indirect associations include (but are not limited to) (1) the influence of the COVID-19 pandemic on treatments (e.g., service utilization) for neurological or mental health conditions and (2) neurological, psychiatric, and psychological outcomes associated with the COVID-19 pandemic [25].

SRs and MAs must meet the following criteria: (i) use of a comprehensive search strategy involving two or more electronic databases; (ii) use of an explicit statement describing the inclusion criteria; (iii) use of a formal critical appraisal or quality assessment process for all included studies and report the outcome of that process; and (iv) report findings on outcomes of interest using details on the study and patient characteristics of three or more studies and provide the direction of the findings from any pooled analyses (narrative or MA) carried out, including direction of effect and any statistical significance.

Eligible SRs or MAs of psychiatric diagnoses must be validated by psychiatrists or by semi-structured interviews relying on standard diagnostic criteria (i.e., the Diagnostic and Statistical Manual of Mental Disorders or the International Classification of Diseases). Similarly, the neurological diagnoses are coded according to the International Classification of Diseases or any standard alternative coding system.

The subgroup analyses conducted in the original included SRs and MAs are considered and reported in the current umbrella review. The mental health response to the COVID-19 pandemic examines the following populations, including but not limited to (1) medical staff, (2) people with mental disorders, (3) people with neurological disorders, (4) people with other physical diseases, (5) children and adolescents, and (6) elderly people. The stressors-associated mental health responses are also explored, including (1) fear of infection (e.g., self or family), (2) infection control measures (e.g., stay-at-home, lockdown), (3) societal changes, and (4) economic impact (e.g., loss of job/income). If possible, the impact of different COVID-19 variants (e.g., alpha, beta, gamma, delta) are explored whenever available on the eligible SR and/or MAs.

We select SRs and MAs of observational studies (case-control, cohort, cross-sectional, or ecological studies). When data are incomplete, the corresponding author is contacted and invited to send additional information. We only include SRs and/or MAs reporting associations, whilst ones reporting only prevalence or incidence rates are excluded. Whenever two or more SRs and/or MAs are available reporting on the same association, we select the one with the largest number of included studies. If there are SRs and MAs with the same number of included studies, then we select the most recent one. Finally, as a third criterion, we select the one with the highest overall methodological quality (vide infra).

### 2.3. Quality of Evidence

Two independent investigators assess the methodological quality of SRs and MAs by using A Measurement Tool for the Assessment of Multiple Systematic Reviews (AMSTAR 2) to quantify the methodological and content quality [26]. AMSTAR 2 includes assessment of study eligibility criteria, identification and selection of studies, data collection methods, study appraisal methods and findings, and synthesis methods. The methodological quality is categorized into low (<4), medium (4–7), and high (>7). Content quality is categorized into low (<4), medium (4–6), and high (>6). The lowest score between methodological and content quality determines the overall quality. Any disagreements are solved by consensus.

Many systematic reviews and meta-analyses focusing on a similar topic include a different number of primary studies; the overall results and conclusions of an umbrella review can therefore be biased. To assess the potential impact of the overlap in the inclusion of the same primary studies, the degree of overlap within and between reviews are measured using the validated corrected cover area (CCA) method [27]. A CCA score of 0–5 indicates slight overlap, 6–10 moderate, 11–15 high, and >15 very high [27]. Each SRs and MAs with sufficient evidence and no hints of bias are reassessed after examination of any data errors and the eligibility of each study. After that, we then appraise the evidence by using the Grading of Recommendations Assessment, Development and Evaluation tool [28].

### 2.4. Data Extraction

All data are re-extracted from each primary study onto piloted forms. One author performs the primary data extraction, and this is independently reviewed by at least one other author. Disagreements are resolved through consensus. The original, primary publication for each included review is used for data extraction.

The data extracted includes specific details about the included studies (e.g., study population) and the review methods (e.g., number of databases searched, search date, and any date, location, nor language restrictions on the search). Patient characteristics (e.g., age, sex, history of physical illness) are also extracted from the included review, if reported. Where results are presented for pooled outcomes and subgroups, we extract pooled outcomes to maximize sample size. If results are presented across several different measures on the same outcome, effect sizes are averaged across all reported assessment measures, to avoid bias associated with selective preference for significant results. The authors’ overall conclusion or recommendation is also extracted.

If both unadjusted and adjusted associations are available, we extract the adjusted effect size because the latter adjusted associations attempt to address confounding as best as possible. If only unadjusted relationships are available, we extract these and plan a sensitivity analysis to exclude such studies. Where data are only available in graphical format, we use WebPlot to digitize the data into an extractable format.

### 2.5. Primary and Secondary Outcomes

The primary outcomes will include the direct and indirect associations between COVID-19 and neurological, psychiatric, and psychological implications. Studies are required to summarize the clinical symptoms using continuous outcomes or binary outcomes to be included (e.g., change in depression rating scales, risk ratio of sleep disorder). Studies that only addressed outcomes in a narrative way without any assessment of clinical status are excluded. The secondary outcomes will include the subgroup analyses conducted according to the categorical moderating variables (e.g., specific populations, specific stressor-related mental health responses, and COVID-19 variants).

### 2.6. Statistical Analysis

A tabular summary of review characteristics (e.g., year of publication, months of data collection, county of origin, geographical regions, respective waves of the pandemics, number of included studies, settings, design of included studies, and other moderators, and/or context as provided in the included SR and/or MAs) is reported. We convert continuous non-standardized outcomes, such as weighted mean differences, to standardized mean differences, and binary outcomes to the odds ratio with 95% confidence intervals. Whenever data conversion is not be possible, we maintain the original effect sizes as reported in the original SRs and/or MAs. Whenever the data are derived from MAs with fixed-effects models, we recalculate the effect sizes using random-effects models. Random-effects models are used because we anticipate considerable heterogeneity in methodology, outcome definitions, and sample characteristics. We calculate the 95% prediction intervals for the summary estimates based on random-effects modelling. This statistic represents the range in which the effect estimates of future studies examining the same associations will fall [29].

We extract the degree of heterogeneity across studies using the *p* value of the chi-squared based Q test and the I^2^ metric of inconsistency [30]. For the Q statistic, *p* < 0.05  is considered significant. The I^2^ metric ranges between 0% and 100%. The degree of heterogeneity is defined as substantial (I^2^ > 50%) and considerable (I^2^ > 75%).

We examine whether smaller studies provide higher side effects estimates than larger studies, which is an indication of publication bias, true heterogeneity, or chance [31,32]. An indication of small study effects is evaluated by the Egger’s regression asymmetry test (*p* < 0.10) and whether the random effects summary estimate is larger than the point estimate of the largest study in each association. We assess excess significance bias by evaluating whether the observed number of studies with nominally statistically significant results (“positive” studies, *p* < 0.005) in the published literature are different from the expected number of studies with statistically significant results [33]. The expected number of statistically significant studies in each association is calculated from the sum of the statistical power estimates for each component study using an algorithm from a non-central t distribution [34,35,36]. The power estimates of each component study depends on the plausible effect size for the tested association, which is assumed to be the effect of the largest study (that is, the smallest standard error) in each association [27,36]. Excess significance for individual meta-analyses is determined at *p* < 0.10. In addition, we extract any adjustment for publication bias as reported in the original meta-analytic studies.

For SRs that do not provide a meta-analytic estimate, we annotate the main conclusion of the study and also take note of the reasons reported by the authors for not conduction a quantitative summary of the results.

### 2.7. Credibility of Evidence

We use the following criteria to assess the credibility of the evidence of observational studies. On the basis of previously proposed criteria considering the amount of evidence, statistical significance, heterogeneity, small-study effect, excess significance, and PI [29,37,38,39]. The classification rules include: (1) having *p* < 10^−6^ on the basis of the random-effect model; (2) having >1000 participants in a single meta-analysis; (3) having low or moderate heterogeneity (I^2^ < 50%); (4) having 95% PI that excludes the null value; (5) having no evidence of small-study effect; (6) having no evidence of excess significance bias. The meta-analyses of observational studies with summary results of *p* < 0.05 are classified into four categories. Meta-analysis that meets criteria (1)–(6) is classified as convincing evidence (not suggestive of bias; class I evidence); meta-analysis that meets criteria (1)–(4) is classified as highly suggestive evidence (class II evidence); meta-analysis that meets criteria (2) and has *p* < 0.001 is classified as suggestive evidence (class III evidence); meta-analysis that has only *p* < 0.05 ais classified as weak evidence (class IV evidence). The classification rules are detailed in Table 1.

## 3. Discussion

In this umbrella review, we will undertake a comprehensive review of previously published SRs and/or MAs assessing the direct and indirect associations between COVID-19 and neurological, psychiatric, and psychological outcomes. Using evidence from this review, we expect to examine which neuro-psycho-psychiatric and psychological implications of COVID-19 are supported by the highest quality of evidence. We anticipate that the methodological quality of most included SRs and/or MAs will fall short from optimal standards [40]. The results of our UR will be of interest to clinicians, policymakers, and patients. However, findings from the present protocol should be considered in several limitations. First, studies related to some topics which are not yet synthesized on systematic reviews and meta-analyses might not be captured by this umbrella review and comprises a potential limitation of this work. Second, umbrella reviews focus on existing MAs; therefore, SR with no meta-analytic approach are excluded. Therefore, generalizability and the final results should be interpreted with caution. Additionally, umbrella reviews do not appraise the quality of all the individual studies included in each meta-analysis, so we will conduct the umbrella analysis and then to determine the necessity of sensitivity analysis based on the quality level of AMSTAR 2. Finally, the poor quality of rapid and poorly transparent protocols for the systematic reviews and/or meta-analyses included in our study may also limit the generalizability of some of the reported associations.

## 4. Conclusions

This UR will be among the first to systematically explore and integrate the highest level of evidence available on bidirectional associations of the COVID-19 pandemic with neurological, psychiatric, and psychological outcomes. The findings of our study will provide relevant evidence from a public health perspective on the neurological, psychiatric, and psychological implications of the ongoing COVID-19 pandemic. Some of our findings may also have health policy implications within the context of possible upcoming pandemics.

## Figures and Tables

**Table 1:** Credibility assessment criteria.

Evidence Classification	Detailed Criteria
Convincing (class I)	Associations with *p* < 10^−6^; having >1000 participants in a single meta-analysis; having low or moderate heterogeneity (I^2^ < 50%); having 95% PI ^1^ that excludes the null value; having no evidence of small-study effect; having no evidence of excess significance bias.
Highly suggestive (class II)	Associations with *p* < 10^−6^; having >1000 participants in a single meta-analysis; having low or moderate heterogeneity (I^2^ < 50%); having 95% PI that excludes the null value
Suggestive (class III)	Associations with *p* < 0.001 and having >1000 participants in a single meta-analysis.
Weak (class IV)	Remaining statistically significant associations with *p* < 0.05

^1^ PI = prediction interval.

## Data Availability

Not applicable.
